# Healthy Lifestyle Recommendations: Do the Beneficial Effects Originate from NAD^+^ Amount at the Cellular Level?

**DOI:** 10.1155/2020/8819627

**Published:** 2020-12-12

**Authors:** Borut Poljsak, Vito Kovač, Irina Milisav

**Affiliations:** ^1^Laboratory of Oxidative Stress Research, Faculty of Health Sciences, University of Ljubljana, Zdravstvena pot 5, Ljubljana, Slovenia; ^2^Faculty of Medicine, Institute of Pathophysiology, University of Ljubljana, Zaloska 4, Ljubljana, Slovenia

## Abstract

In this review, we describe the role of oxidized forms of nicotinamide adenine dinucleotide (NAD^+^) as a molecule central to health benefits as the result from observing selected healthy lifestyle recommendations. Namely, NAD^+^ level can be regulated by lifestyle and nutrition approaches such as fasting, caloric restriction, sports activity, low glucose availability, and heat shocks. NAD^+^ is reduced with age at a cellular, tissue, and organismal level due to inflammation, defect in NAMPT-mediated NAD^+^ biosynthesis, and the PARP-mediated NAD^+^ depletion. This leads to a decrease in cellular energy production and DNA repair and modifies genomic signalling leading to an increased incidence of chronic diseases and ageing. By implementing healthy lifestyle approaches, endogenous intracellular NAD^+^ levels can be increased, which explains the molecular mechanisms underlying health benefits at the organismal level. Namely, adherence to here presented healthy lifestyle approaches is correlated with an extended life expectancy free of major chronic diseases.

## 1. Introduction

Fasting, caloric restriction, sports activity, low glucose availability, and heat shocks are lifestyle and nutrition approaches that influence NAD^+^ levels [1–6]. Deficiency in NAMPT-mediated NAD^+^ biosynthesis, increased inflammation, and the PARP-mediated NAD^+^ depletion are causes of reduced NAD^+^ levels with age at a cellular, tissue, and organismal level [7, 8].

Coenzyme nicotinamide adenine dinucleotide (NAD^+^), which contains two covalently joined mononucleotides (nicotinamide mononucleotide or NMN, and AMP) [9], has an important role in an energy metabolism like mitochondrial electron transport, glycolysis, and citric acid cycle [10] in order to generate adenosine triphosphate (ATP) [11]. NAD^+^ is also a rate-limiting substrate for many signalling enzymes such as sirtuin (SIRT) proteins SIRT1 and SIRT3, the poly (ADP-ribose) polymerase (PARP) proteins PARP1 and PARP2, a COOH-terminal binding protein (CtBP), cyclic ADP-ribose (ADPR) synthetases CD38 and CD157, and many other NAD^+^-dependent enzymes. These enzymes are involved in important cellular processes, like DNA repair, stress response, genomic stability, chromatin remodelling, circadian rhythm regulation, cell cycle progression, insulin secretion and sensitivity, and expression of the inflammatory cytokines, thus translating changes in energy status into metabolic adaptations [12]. NAD^+^ is recycling during ATP formation in processes of glycolysis, beta-oxidation, Krebs cycle, and electron transport in cytosol and mitochondria and shifts between reduced and oxidized forms as required for the continuous flow of electrons across the metabolic pathways. Therefore, the NAD^+^ molecule is conserved during these processes. On the other hand, the NAD^+^ is consumed during cellular signalling, in adenosine diphosphate (ADP)-ribosyl transfer reactions, by poly-ADP-ribose polymerases (PARPs), sirtuin deacetylases (Sirtuins), and the cluster of differentiation 38 (CD38), i.e., the nicotinamide (NAM) unit is separated. NAD^+^ half-life is between 1–2 h in the cytoplasm and nucleus and approximately 8 h in the mitochondria [13] and can be salvaged and reused by three pathways: (1) *de novo* synthesis (from L-tryptophan), (2) Preiss-Handler pathway (from nicotinic acid or nicotinic acid ribose), and (3) salvage pathway (from niacinamide/nicotinamide, nicotinamide riboside, and nicotinamide mononucleotide) [9, 14–18]. NAD^+^ is mainly produced by the NAD^+^ salvage pathway where nicotinamide phosphoribosyltransferase (NAMPT) is the rate-limiting enzyme, converting NMN into NAD^+^ [19–21]. NAMPT regulates processes related to the pathological processes of obesity and a type 2 diabetes mellitus by influencing lipid and glucose metabolism, insulin resistance, the oxidative stress response, apoptosis, and inflammation [22, 23].

**Figure 1:**
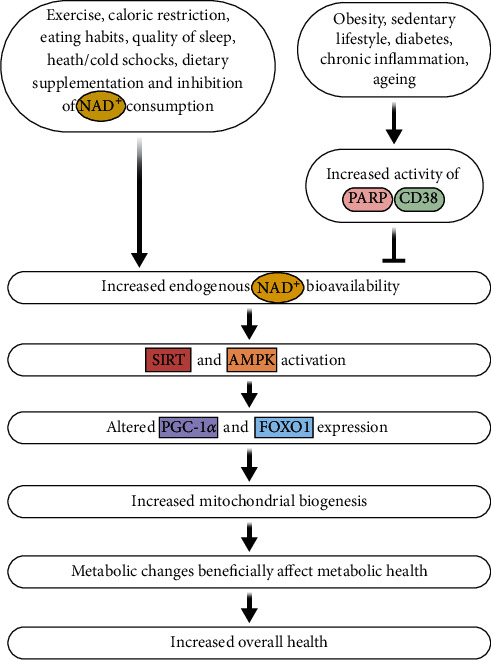
Health benefits as a result of implementing approaches to increase NAD^+^ bioavailability.

The NAD^+^/NADH ratio influences also the reactive oxygen species (ROS) and oxidative stress formation through regulation of intracellular ATP production, different metabolic enzymes, and redox state. An increase of NAD^+^ and/or NAD^+^/NADH ratio can increase cell defence, can induce DNA repair and apoptosis through activation of PARPs and sirtuins, and thus plays an important role in the prevention of cancerogenesis and many other diseases [14, 24]. For example, cellular NAD^+^/NADH ratio regulates SIRT1 enzymatic activity, which further regulates a number of target proteins [25], such as FOXO family of transcription factors [26–28], p53 [29, 30], PGC-1a [31, 32], and NF-kB [33–35]. While chronic diseases and ageing are related to decreased NAD^+^ levels [16, 36, 37], different lifestyle factors have been found that ameliorate NAD^+^ bioavailability, which positively affects SIRT stimulation and subsequent PGC-1*α* and FOXO1 expression, leading to mitochondrial changes and metabolic adaptations (Figure 1) [38]. Increased available cellular energy, improved stem cell and mitochondrial function, DNA repair [39], telomere maintenance [40], and enhanced metabolic activity are prerequisites for effective health span and life span [41, 42] as demonstrated by studies where NAD^+^ levels were intentionally increased [23, 43–48].

## 2. Caloric Restriction, Eating Habits, and NAD^+^ Levels

A well-balanced diet in macro- and micronutrients represents a basis for health and well-being. Limited calorie intake continues to be the strategy supported by the greatest evidence for ensuring increased lifespan and health [49]. In different model organisms, a significant increase in lifespan was reported if calories were restricted between 25–60% relative to normally fed control [50, 51]. How is caloric restriction connected with NAD^+^ levels? CR stimulates the NAD^+^ salvage pathway leading to increased NAD^+^ bioavailability by activating the expression of NAMPT, which triggers the NAD^+^ salvage pathway by transforming nicotinamide (NAM) to NAD^+^ [52]. Caloric restriction increases NAD^+^ levels, while lowers NADH levels and activates sirtuins [53]. For example, caloric restriction extends the yeast's life span by lowering the level of NADH, since NADH is a competitive inhibitor of Sir2 [54]. Thus, activation of sirtuins with a sufficient amount of bioavailable NAD^+^ is a necessary condition for the life-span extension provided by CR [55, 56]. Specifically, Sirt1 regulates CR by detecting intracellular low energy levels and provoking physiological changes relevant to health and longevity [57]. On the other hand, inactivation of SIRT1 results in the prevention of CR-mediated lifespan extension [58].

Studies on caloric restriction revealed that it is more important to improve the ratio between NAD^+^ and NADH than to raise the overall amount of cellular NAD^+^ [59]. Namely, caloric restriction reduces NADH amount more than it influences the NAD^+^ levels, at least in yeast [54, 60]. It seems that lowering NADH is an important factor responsible for the increased activity of the NAD^+^-consuming enzymes, as NADH is an inhibitor of Sirtuins and PARPs [54].

Besides by caloric restriction, NAD^+^ levels can be increased with food and commercially available supplements. Ingestion of the amino acid tryptophan or forms of vitamin B3 (niacin, nicotinic acid, niacinamide) as well as nicotinamide riboside (NR), nicotinamide mononucleotide (NMN), and nicotinic acid riboside (NaR) stimulates the formation of NAD^+^ [61–64]. Daily requirements for NAD^+^ synthesis can be obtained either with dietary tryptophan or with around 15 mg/d of daily niacin, a collective term for nicotinic acid (NA) and nicotinamide (NAM) [61], which can be found in meat, fish, and dairy products [65].

Small-scale human clinical studies have shown that NAD^+^ boosters such as NMN, NR, and niacin can increase the levels of NAD^+^ in volunteers and are relatively safe for human consumption [6, 66–72]. Most of the side effects reported during treatment with NAM, NR, and NMN are minor (e.g., diarrhea, nausea, rashes, hot flashes, cramps in the legs, erythema) and occur relatively rarely [73, 74]. Increased acetylcarnitine concentrations in skeletal muscle and minor changes in body composition and sleeping metabolic rate were reported in the recent study on NR supplementation in healthy obese humans [75]. The evidence for assessing the health risk is still limited, and long-term exposure to NAD^+^ booster (NR, NMN) has not yet been investigated in human clinical trials or human clinical trials are not yet completed. In addition, there is insufficient data on increasing the levels of NAD^+^ in various clinical disorders.

As data for some newly discovered NAD^+^ precursor forms are scarce, NAD^+^ supplements should be tested in a manner similar to drugs in development [72]. Niacin equivalents/precursors are found in animal and plant foods, mainly in the form of NA and NAM. Additionally, recently discovered NAD^+^ intermediates, such as NMN and NR, are also in foods, like cucumber, cabbage, and immature soybeans. Broccoli has 0.25–1.88 mg of NMN per 100 g, avocado and tomato 0.26–1.60 mg/100 g. Much less NMN can be found also in raw beef and shrimp (0.06–0.42 mg/100 g) [45] as well as human and cow milk at micromolar concentrations [76, 77]. NAD^+^ biosynthesis can be increased by direct activation of NAD^+^ biosynthetic enzymes by several AMPK and NAMPT activators, like nonflavonoid polyphenol resveratrol, metformin, 5-aminoimidazole-4-carboxamide ribonucleotide, P7C3, leucine, epigallocatechin gallate, and proanthocyanidins [78–86]. CD38, its homologue CD157, and PARP-1 inhibitors could additionally increase NAD^+^ availability; however, they are registered as medical drugs for cancer treatment [24], thus beyond the scope of this review.

## 3. Eating Habits

NAD^+^/sirtuin pathway could be influenced also with nutritional approaches, e.g., eating habits. At what time and what and how much food we eat influence intracellular NAD^+^ bioavailability by altering electron transport in mitochondria. For example, a high-fat/sugar diet causes energy overload, culminating in reduced NAD^+^/NADH ratio [87] and decreases NAD^+^ levels [23, 63]. Also, a low AMP/ATP ratio causes a decrease in NAD^+^ or NAD^+^/NADH, in situations when enormous amounts of calorically rich food (lipids and/or carbohydrates) are eaten. This additionally leads to elevated blood sugar and insulin levels, increased NADH/NAD ratio, and increased formation of ROS, which triggers the postprandial oxidative stress and oxidative damage [88–91]. Large amounts of electrons from sugars enter the mitochondria after a large portion of food that generates more superoxide at complex 1 (NADH: ubiquinone oxidoreductase) and complex III (ubiquinol: cytochrome c oxidoreductase) [92]. Efficient electron flow and avoidance of electron leaks (superoxide formation) can be achieved if ATP is regularly consumed; for example, by moderate sport activity or any kind of physical work. This increases the AMP/ATP ratio and NAD^+^ availability [87, 93, 94]. The link between the metabolism and NAD^+^ is further strengthened by observations that besides overnutrition, tissue NAD^+^ levels decrease also with high-fat diets and obesity [23, 63, 95–98]. Rappou et al. [99] compared SIRT1, SIRT3, SIRT7, and NAMPT expressions and total PARP activity in lean and obese subjects. Results indicated lower sirtuins and NAMPT expressions and increased total PARP activity in obese compared to lean subjects. After a moderate weight loss, SIRT1 and NAMPT expressions increased while PARP activity significantly decreased in subjects upon the weight loss. Similar results were obtained in healthy men during lipid overfeeding [100]. Other studies observed that obesity is associated with low NAD^+^ levels or SIRT pathway expression [101]. On the other hand, supplementation with NAD^+^ precursors or intermediates activates sirtuins and oxidative metabolism resulting in the protection against high-fat diet-induced obesity [63], improved glucose tolerance and hepatic insulin sensitivity [23], and lipid metabolism [45].

A high-fat caloric diet induces obesity through the protein CD38, which is a regulator of body weight and an NAD^+^ consumer [102]. Mice deficient in CD38 are protected against the high-fat diet-induced obesity due to boosted metabolic rate in part via a NAD-dependent stimulation of SIRT-PGC1alpha axis [102]. Adipose tissue elevates the expression of CD38 and inflammation-related genes in obese people [103, 104]. In line, low expression of CD38 protected against obesity when fed a high-fat diet in animals [102, 105].

NAD^+^ level is not only nutritionally controlled, but it depends also on the sports activities and other lifestyle factors.

## 4. Exercise and NAD^+^ Levels

Physical activity and exercise, as part of a healthy lifestyle, have a significant impact on health outcomes, including improved motor skills, healthy bones, enhanced aerobic fitness, efficient heart and lung function, improved cardiovascular health, lowered risk of stroke, certain types of cancer and diabetes, improved metabolic flexibility and mitochondrial function, and a positive effect on cognitive function and mental health—including on depressive symptom improvement and anxiety- or stress-related disease [38, 106–108].

How does sports activity affect NAD^+^ levels? Aerobic exercise training or any kind of exercise/sports activity increases the amount of NAD^+^ due to the induction of skeletal muscle's NAMPT expression that was shown in rodent and human studies [109–111]. Namely, in human skeletal muscle, exercise training reverses the age-dependent decline of NAD^+^ by stimulating the NAD^+^ salvage pathway, in which nicotinamide NAMPT is a rate-limiting enzyme [112]. Exercise and aerobic sports activity increases the amount of NAD^+^ due to the induction of skeletal muscle's NAMPT expression [109] and reverses the age-dependent decline of NAD^+^ by stimulating the NAD^+^ salvage pathway [112] through the 5′ AMP-activated protein kinase (AMPK) pathways [4].

NAD^+^ has an important role in the generation of intracellular ATP, which is required for exercise and sports activities. On the other hand, as already mentioned, ATP production in mitochondria represents the main source of free radical generation. The reduction state of complex I in mitochondria depends strongly on the NAD^+^ and NADH levels. Ameliorating the NAD^+^/NADH ratio by elevated ATP consumption (e.g., sports activity) or decreased ATP production (e.g., intermitted fasting, consumption of small portions of food, and CR) regulates the magnitude of superoxide-generation from the transfer of electrons to molecular oxygen at mitochondrial complexes I and III and can thus ameliorate the intensity of oxidative damage [113]. Increased demand for energy during the exercise is sensed by the cell and activates AMPK, which can modulate NAD^+^ bioavailability [38]. Both exercise and caloric restriction trigger the metabolic stress that follows by adaptation by inducing NAMPT expression through the AMPK [4, 109, 114] resulting in increased NAD^+^ levels available for sirtuins and PARPs.

A recent study by de Guia et al. revealed that different exercise training methods reverse the age-dependent decline in NAD^+^ salvage capacity in the human skeletal muscle [112]. Namely, both aerobic and resistance exercise training increased NAMPT levels in young and older individuals. In aged rats, exercise training also increased NAD^+^, NAMPT levels, and SIRT1 activity [111] and accelerates the *de novo* biosynthesis of NAD^+^ from L-tryptophan [115].

The important function of NAD^+^ during sports activity is its role as a hydrogen/electron transfer molecule for adenosine triphosphate (ATP) production and mitochondrial biogenesis in muscle cells [116]. Additionally, sports activity increases the NAD^+^ amount also at the systemic level [117] that results in health benefits at the organismal level due to the NAD^+^ role in multiple and diverse cellular processes, in addition to redox reactions, such as deacetylation and ADP-ribosylation [116]. During the intense sports activity, ATP is consumed; thus, the need for NADH as the electron donor increases, which in the end results in the boosted formation of oxidised NAD^+^ and decreased NADH, i.e., an improved NAD^+^/NADH ratio. The total amount of NAD^+^ is not significantly changed during the redox reaction; however, the *NAD^+^/NADH* (and NADP to NADPH) ratio is changed in favour of NAD^+^ [61], which activates sirtuins, PARPs, CD38, and other NAD^+^-consuming reactions. Since NAD^+^-consuming enzymes intervene in many crucial cellular processes, many healthy processes at the organismal level are enhanced by the implementation of exercise and sports activity.

Surprisingly, NR, the NAD^+^ precursor, decreases exercise performance in rats [118], most likely due to the pleiotropic metabolic and redox properties of NAD^+^ and NADP+. Nicotinic acid also reduced the capacity for high-intensity exercise in humans [119], which is ascribed to lower plasma free fatty acids, leading to earlier fatigue. Studies on NAD^+^ precursor supplementation implied prevention of vascular dysfunction, oxidative stress, and muscle age-degeneration in mice [45, 46, 120]. Accordingly, it is important to preserve a high NAD^+^ to NAD^+^/NADH ratio that can be achieved also by sports activity.

## 5. Circadian Rhythms, Sleeping Habits, and NAD^+^ Levels

Sleep disorders predispose persons to chronic diseases like obesity, depression, diabetes, and many cardiometabolic diseases, which are significantly associated with mortality and morbidity [121, 122] [123–125]. Contrary, a steady pattern of waking and sleeping is associated with health promotion and longevity [126]. Prolonged disruptions of circadian rhythms are associated with negative health consequences [127]. NAD^+^ levels and sirtuin activity regulate a healthy circadian rhythm of sleep and wakefulness; concurrently, the NAD^+^ level is supervised by circadian rhythm and involved in the circadian clock regulation. NAD^+^ levels oscillate with a 24 h rhythm; these can be modified by feeding and sleeping time [128–130]. The central internal clock is in the hypothalamic suprachiasmatic nucleus, and the circadian rhythms are coordinated by intracellular proteins called “circadian clocks.” These proteins are regulated by a transcriptional negative feedback loop between transcriptional activators CLOCK and BMAL1 and repressors CRY and PER. CLOCK, the core circadian regulator, is a histone acetyltransferase whose activity is outweighed by the nicotinamide adenine dinucleotide- (NAD^+^-) dependent histone deacetylase SIRT1 [131, 132]. CLOCK : BMAL1 heterodimer balances the circadian expression of NAMPT, which regulates the NAD^+^ biosynthesis. The activity of NAMPT is constrained by light—or sleep—deprivation and upregulated by darkness and night [130].

With ageing, NAMPT activity declines, and consequently, NAD^+^ bioavailability drops [7, 133], leading to the deterioration of the circadian rhythm (change in amplitude, period, and phase). Disrupted circadian rhythms were reported in many pathological conditions including cardiovascular diseases, diabetes, cancer, and accelerated ageing [134, 135]. On the contrary, matching the innate circadian period results in health improvements [135–140].

## 6. Environmental Stress: Heat/Cold Shock and NAD^+^ Levels

Exposure to the elevated heat for short time periods can result in beneficial health effects. Cardiovascular responses to long-term adaptations in response to heat stress result in reduced blood pressure and arterial stiffness and improved endothelial and microvascular function [141]. For example, regular sauna bathing may be linked to several health benefits, which include decreased risk of sudden cardiac death and cardiovascular and all-cause mortality [142], reduction in the risk of neurocognitive diseases and nonvascular conditions such as pulmonary diseases, and amelioration of conditions such as arthritis, headache, and flu [143]. What is more, heat stress cardioprotection and improved postischemic functional recovery in the heat-stressed hearts after cardioplegic arrest due to increased NAD^+^ and NADP+ concentrations were observed [144]. Heat shock triggers an increase in the NAD^+^/NADH ratio as a result of decreased NADH levels and an increase in recruitment of SIRT1 to the hsp70 promoter [25]. Enzyme nicotinamide mononucleotide adenylyltransferase (NMNAT), which catalyzes nicotinamide adenine dinucleotide (NAD^+^) synthesis, is elevated during conditions of heat shock and transcriptionally regulated by the heat shock factor (HSF) and hypoxia-inducible factor 1*α* (HIF1*α*) *in vivo* [145, 146].

In addition to heat stress, also cold stress-induced physiological responses and activation of brown adipose tissue (BAT) have health benefits [147]. BAT mainly burns energy in contrast to white adipose tissue (WAT), which stores fat [141]. In mouse and human BAT, cold exposure activates NAD^+^ biosynthesis mediated by a rate-limiting enzyme, NAMPT [148]. BAT is abundant in mitochondria and plays a role in energy expenditure related to producing heat by an energy-dissipating process of nonshivering thermogenesis, leading to changes in lipid metabolism [149] and other health benefits like the absence of low-grade inflammation, increased insulin sensitivity, and decreased liver fat [150, 151]. Degradation, whitening, and impaired function of BAT promotes obesity [152–155].

The facts supporting the “NAD^+^ > SIRTs > positive effect” pathway as the mechanism of action for the beneficial effects of NAD^+^ repletion strategies have been presented so far. Are there indications of concerns about increasing the levels of NAD^+^?

## 7. Potential Deleterious Effects of Increased NAD^+^

As already discussed, NAD^+^ precursors, nicotinic acid (NA), and NR decreased exercise performance in young rats [118] and reduce the capacity for high-intensity exercise in humans [119], although old individuals seem to benefit from NR supplementation. Namely, increased NAD(P)H levels, decreased oxidative stress, and improved physical performance were observed only in the old subjects [156]. Kourtzidis et al. [157] expressed concern that redox agents administered exogenously in healthy young populations (not suffering from antioxidant deficiency) might lead to adverse effects. Nicotinamide (NAM) overdose was reported to cause hepatotoxicity in rare cases [158]. In addition, it was observed that a high dose of dietary NR caused glucose intolerance and dysfunction of the white adipose tissue in mice fed a slightly obesogenic diet [159].

Regarding longevity, overexpression of SIRT1 was found not to extend lifespan in mice fed standard diets, although they had better general health and fewer carcinomas [160]. Mitchell et al. [43] observed that supplementation with NAM in the mouse model did not change the lifespan, in spite of the improved healthspan. Additionally, Chen et al. [161] challenge the paradigm that CR induces SIRT1 activity in all tissues. Similarly, Frederick et al. [162] suggest that NMN and NR increase in NAD biosynthesis is cell- or tissue-specific.

It appears that the NAD^+^ levels could have both procancer and anticancer effects, as NAD^+^ is a critical protective factor in early cancer development and could become a damaging factor later in the phase of cancer progression and promotion. Namely, during cancer promotion, progression, and treatment, increased NAD^+^ levels could have adverse effects on the malignancy process due to increased cell survival, growth advantage, increased resistance to radio- and chemotherapy, and promotion of inflammation. In contrast, NAD^+^ restoration could prevent or reverse the phenotype of malignant cells in the early stages by inducing cellular repair and adaptive stress responses and regulating cell cycle arrest and apoptotic removal of damaged cells (reviewed in [24]). In addition, the compound FK866, which inhibits the nicotinamide recycling enzyme NAMPT, is a tumor apoptosis inducer due to the NAD^+^ depletion [163, 164] and is used as an anticancer drug.

In the area of inflammation/sepsis, there is also controversy regarding the NAD(+)-dependent sirtuin family, as elevated NAD(+) levels play a different role in the different stages of sepsis. In the initial (proinflammatory) phase, which is characterized by a cytokine storm, overproduction of reactive oxygen species (ROS), and metabolic shift [165], SIRT1 activation shows positive effects, whereas the SIRT1 expression should be inhibited in the later stages of sepsis [166]. Therefore, due to the dynamic phases of sepsis, the role of SIRT1 cannot simply be defined as beneficial or detrimental. Increased NAD^+^ might have also negative effects on inflammatory disorders, such as rheumatoid arthritis due to stimulated inflammatory cytokine secretion by leukocytes [167].

Another potential risk could be posed by the toxic degradation products and metabolites of NAD^+^ precursors, e.g., nicotinic acid adenine dinucleotide (NAAD), *N*-methyl nicotinamide (MeNAM), and 2-PY[71, 168]. Lastly, increased NAM levels due to the supplementation with NAD^+^ precursors (NAM, NR, or NMN) could inhibit PARPs and CD38 activities [169], while SIRT1 feedback inhibition *in vivo* by NAM may not be so important [170, 171]. Increased levels of NAM might alter also the methyl pool used to methylate DNA and proteins [171].

## 8. Conclusions

It is not only the NAD/NADH redox role as hydride and electron transfer in redox metabolic reactions but mainly the NAD^+^ as the signalling molecule and substrate for sirtuins and PARPs that is responsible for the health benefits and longevity. Cellular NAD^+^ content and an adequate NAD^+^/NADH ratio can postpone pathologic processes associated with impaired cell signalling and mitochondrial function [87, 172, 173]. Thus, for maintaining optimal cellular functioning and organismal health, it is necessary to implement the lifestyle approaches that stimulate increased NAD^+^ levels. The synergistic effects of different measures to ensure a healthy lifestyle are important, as there is an intimate and reciprocal relationship between them. For example, sedentary lifestyle, overeating, and excessive intake of fat and sugar are associated with disturbances in circadian rhythms [174, 175] and downregulation of NAMPT gene expression [4]. Implementation of the time-restricted feeding without reducing the caloric intake (8 h per day feeding/16 h per day fasting) improved the robustness of circadian and metabolic rhythms and prevented metabolic diseases in mice on a high-fat diet [176]. Lifestyle approaches, such as exercise and CR, can reverse insulin resistance and type 2 diabetes mellitus (T2DM) [12]. Both manipulations increase the NAMPT-mediated NAD^+^ generation, activate mechanistic pathways of AMPK, and enhance the SIRT1 activity and mitochondrial function [4, 114, 177, 178]. Sirtuins affect various cellular processes, including lipid metabolism, insulin secretion, and sensitivity [179]. NAD^+^ levels within cells are regulated by its precursors' intake, biosynthetic pathways, and degradative enzymes [180], which can be additionally balanced by selected lifestyle factors discussed here. In order to provide sufficient NAD^+^ bioavailability and appropriate expression of NAMPT, it is necessary to ingest sufficient amounts of NAD^+^ precursors/intermediates in the vitamin B3 forms, preferably as a part of a normal diet, to practice regular and moderate sports activity, and to observe time intervals between darkness and light exposure as well as the appropriate time intervals between feeding and fasting.

The presented studies support the hypothesis that maintaining NAD^+^ levels leads to healthy cell metabolism, which is beneficial in terms of amelioration of metabolic diseases and ageing. It should be stressed that NAD^+^ is not the only factor, but rather one of the several components that influence cell health. There are many other positive effects of calorie restriction, eating habits, exercise, circadian rhythms, and environmental stress on human health that are beyond the scope of this paper. Although many animal studies have shown the link between NAD^+^ and healthspan, the complex role of NAD^+^ in the etiology of ageing and age-related chronic diseases in humans should be further elucidated. The current state of knowledge about NAD^+^ positive effects on ageing and healthspan is mainly based on experiments on cell cultures and model organisms, so that the positive health effects of NAD^+^ in humans will need to be confirmed in future in-depth studies and clinical trials.
